# Skeletal Fluorosis in a Patient With Computer Cleaner Inhalant Abuse

**DOI:** 10.7759/cureus.37229

**Published:** 2023-04-06

**Authors:** Evan Chen, John Jayman, Nora Bedrossian

**Affiliations:** 1 Internal Medicine, Los Angeles County+University of Southern California (USC) Medical Center, Los Angeles, USA; 2 Internal Medicine, Virginia Commonwealth University (VCU) Medical Center, Richmond, USA; 3 Endocrinology, Los Angeles County+University of Southern California (USC) Medical Center, Los Angeles, USA

**Keywords:** substance use disorder, secondary hyperparathyroidism, computer inhalant abuse, diffuse sclerosis, skeletal fluorosis

## Abstract

Skeletal fluorosis is a metabolic bone disease caused by excessive consumption of fluoride from fluoride-contaminated water or foods. Such a condition often takes place in developing countries without proper handling of drinking water or food. However, in recent years, multiple cases of skeletal fluorosis have been observed in the United States due to the increasing frequency of recreational substance abuse. In this case report, a 26-year-old male with a history of polysubstance use disorder presented to the emergency department after being assaulted by store employees when attempting to steal computer cleaner inhalants. On evaluation for acute traumatic injury, he was incidentally found to have diffuse sclerosis of all visualized bones on knee, femur, and hip X-rays. Labs were significant for elevated serum alkaline phosphatase levels, secondary hyperparathyroidism, and hypovitaminosis D. Given the patient’s history of computer cleaner inhalant misuse and imaging findings, serum and urine fluoride levels were obtained and supported the diagnosis of skeletal fluorosis. Skeletal pain and diffuse sclerosis on imaging should prompt clinicians to include skeletal fluorosis in the differential diagnosis. Cessation of substance use is the primary treatment of fluorosis in the setting of computer cleaner inhalant abuse. However, clinical symptoms and laboratory and imaging abnormalities may take decades to resolve due to the prolonged half-life of fluoride in bone. Proper hydration is crucial, as nephrolithiasis and hypercalciuria have been described during the skeletal unloading of fluoride.

## Introduction

Skeletal fluorosis can be caused by excessive consumption of fluoride from fluoride-contaminated water or foods. Such a condition is endemic in developing areas of countries such as India and China [1-2]. Over-ingestion of fluoride may alter bone development, remodeling, and metabolism by increasing osteoblast activity and periostitis, leading to back pain, bone tenderness, joint immobility or deformity, and arthralgias [1-4]. Complications include pathologic bone fractures, osteoporosis, and osteomalacia [1-3]. Few case reports have presented rare cases of skeletal fluorosis in the United States from recreational substance misuse, such as computer cleaner inhalants, fluoride-containing air dust cleaner, and surreptitious excessive toothpaste consumption [1-3,5-6].

## Case presentation

A 26-year-old male with a past medical history of polysubstance use disorder (including computer cleaner inhalant, methamphetamine, fentanyl), major depressive disorder, unspecified mood disorder, and homelessness presented to the ED after being assaulted by store employees when attempting to steal a computer cleaner inhalant. He was placed on an involuntary psychiatric hold after reporting suicidal ideation. Per the patient, he was hit in the face, abdomen, left hip, and left leg during the assault. He endorsed diffuse bone pain (hips, knees, ankles), insomnia due to inhalant use, and active auditory and visual hallucinations, which he stated had been a longstanding problem for him. The patient reported that he had been inhaling more than 10 bottles of computer cleaner inhalant per day for two years, using methamphetamine and fentanyl every two weeks, and smoking one cigarette per week. He denied a history of childhood fractures but did have a history of traumatic left-sided displaced angle fracture of the mandible status post open reduction, internal fixation with oral maxillofacial surgery as well as a prior minimally displaced right medial malleolus fracture which occurred when he was hit by a car while intoxicated. The patient denied a family history of bone, thyroid, or parathyroid disease and had no known drug allergies. He reported taking three different psychiatric medications in the past but was nonadherent and unable to recall their names.

**Figure 1:**
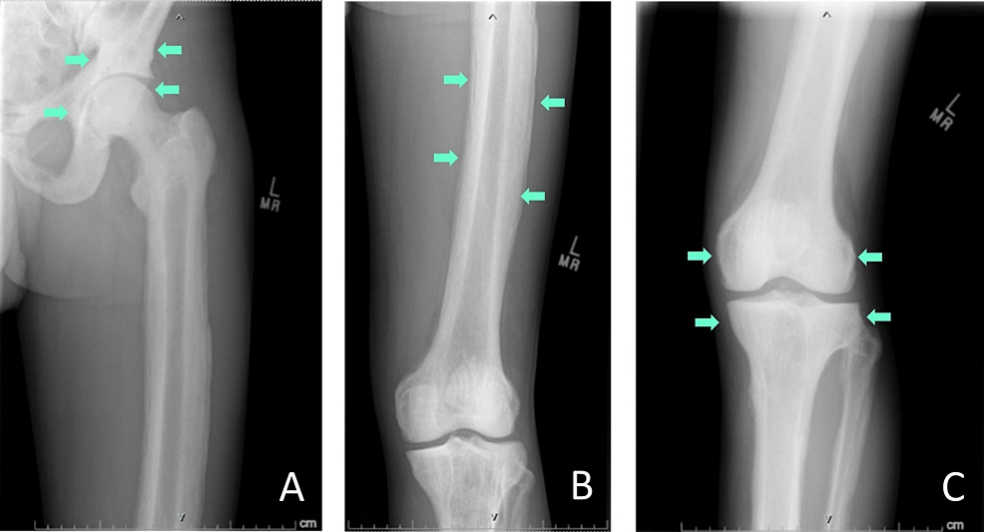
X-rays of the left hip (A), left femur (B), and left knee (C) showed diffusely increased sclerosis and cortical expansion of all visualized bones

**Figure 2:**
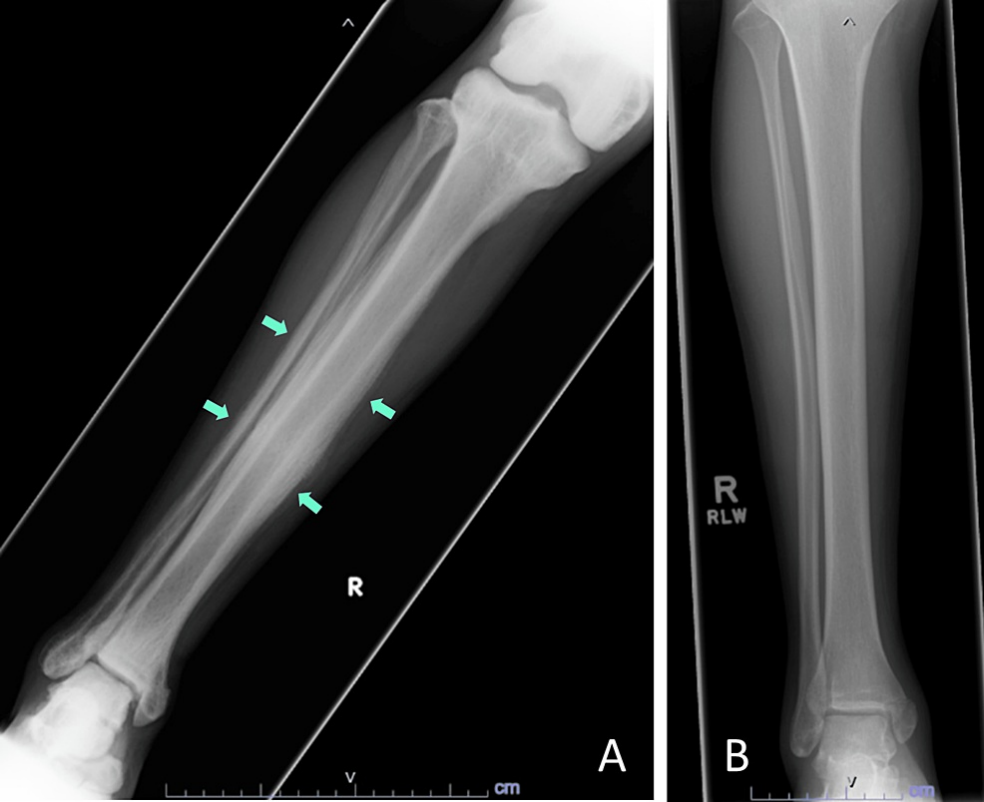
X-ray of the right tibia and fibula (A) showed diffusely increased sclerosis and cortical expansion, while an X-ray from two years prior (B) demonstrated only minimally displaced transverse fracture through the medial malleolus after a motor vehicle accident; no sclerosis or cortical expansion of bones were observed at that time.

**Figure 3:**
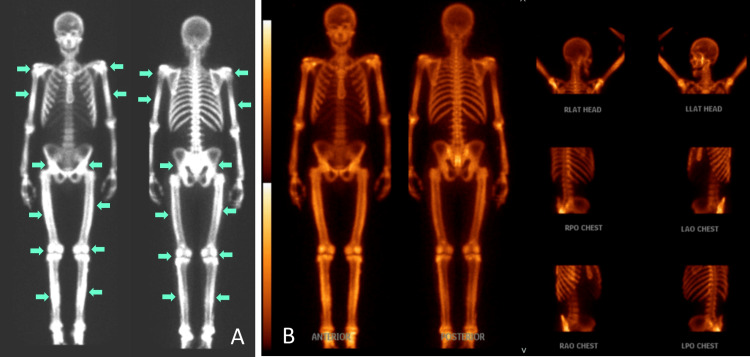
Nuclear medicine bone imaging of the whole body showed diffusely increased lower greater than upper extremity uptake

On arrival at the ED, the patient was afebrile, normotensive, and tachycardic to 130 beats per minute. Physical examination was remarkable for mild ecchymosis under the left orbit, tachycardia, and abrasion over the left hip. Laboratory results were significant for low hemoglobin (11.1 g/dL), mildly low calcium (8.1 mg/dL), significantly elevated alkaline phosphatase (1504 U/L), and normal albumin (4.2 g/dL) (Table 1). Computed tomography (CT) of the cervical spine showed diffuse sclerosis of the visualized axial and appendicular skeleton. The X-rays of bilateral lower extremities showed diffuse sclerosis and cortical expansion of all visualized bones compared to X-rays from two years prior (Figures 1, 2). A nuclear medicine bone scan for further evaluation of sclerosis showed diffusely increased lower greater than upper extremity uptake (Figure 3). Further laboratory evaluations showed a low 25-hydroxyvitamin D level (14 ng/mL), elevated parathyroid hormone (131 pg/mL), and normal thyroid stimulating hormone (0.68 uIU/mL). The patient was given fluid boluses and pain medications. Toxicology was consulted and recommended symptomatic management for inhalant withdrawal and serial electrocardiograms (EKGs) due to potential dysrhythmias from myocardial sensitization secondary to inhalant use. Endocrinology was consulted due to concern for primary hyperparathyroidism and the imaging findings. A diagnosis of diffuse skeletal fluorosis was highly suspected given the patient’s history, laboratory, and imaging findings. The elevated parathyroid hormone level was thought to be due to secondary hyperparathyroidism from hypocalcemia secondary to fluorosis. Serum fluoride and urine fluoride levels were elevated to 0.40 mg/L and 16.88 mg/L, respectively. The C-telopeptide level was normal at 1032 pg/mL. The patient was started on ergocalciferol 50,000 IU daily for three days, followed by cholecalciferol 4000 IU daily for hypovitaminosis D. He was later discharged to a shelter with a substance abuse program after discontinuation of psychiatric hold, resolution of tachycardia, and improvement of symptoms. He was referred to follow up with an in-network endocrinologist and was counseled to maintain abstinence from computer cleaner inhalation and other substance use.

**Table 1:** Relevant laboratory findings ^a^Low level, ^b^Elevated level

Tests	Result	Reference Range
White blood cells (WBC), K/cumm	8.5	4.5-10.0
Hemoglobin (Hgb), g/dL	11.1^a^	13.5-16.5
Hematocrit (Hct), %	34.8^a^	40.0-49.0
Platelets (Plt), K/cumm	464	160-360
Sodium (Na), mmol/L	137	135-145
Potassium (K), mmol/L	4.5	3.5-5.1
Chloride (Cl), mmol/L	102	100-110
Bicarbonate (CO2), mmol/L	20	20-30
Blood urea nitrogen (BUN), mg/dL	8	8-22
Creatinine (Cr), mg/dL	0.45^a^	0.50-1.30
Calcium (Ca), mg/dL	8.1^a^	8.5-10.3
Phosphorus, mg/dL	3.7	2.5-4.5
Aspartate transaminase (AST), U/L	12	10-50
Alanine transaminase (ALT), U/L	22	10-50
Alkaline phosphatase (ALP), U/L	1504^b^	40-129
Total bilirubin, mg/dL	0.6	<1.0
Albumin, g/dL	4.2	3.5-5.0
Gamma-glutamyl transferase (GGT), U/L	23	8-61
Folate, ng/mL	10.5	>=4.6
Vitamin B12, pg/mL	335	232-1245
Vitamin D 25-hydroxylase, ng/mL	14^a^	30-100
Total protein, g/dL	6.3	6.0-8.0
Thyroid-stimulating hormone (TSH), uIU/mL	0.68	0.27-4.20
Parathyroid hormone (PTH), pg/mL	131^b^	15-65
Ammonia, umol/L	26	16-60
Ethanol, mg/dL	<11	<=10
Salicylate, mg/dL	<2.0	<2.0
Acetaminophen, mcg/mL	<5.0	<5.0
Ferritin, ng/mL	52	30-330
Iron saturation, %	9^a^	15-50
Total iron binding capacity (TIBC), mcg/dL	276	250-430
Iron, mcg/dL	25^a^	59-158
C-telopeptide, pg/mL	1032	87-1200
Urine fluoride, mg/L	16.88^b^	0.20-3.20
Serum fluoride, mg/L	0.40^b^	<0.13
HIV antibody-antigen screen	Non-reactive	Non-reactive
Rapid plasma antigen (RPR)	Non-reactive	Non-reactive
Hepatitis A IgM antibody	Non-reactive	Non-reactive
Hepatitis B Core antibody	Non-reactive	Non-reactive
Hepatitis B Surface antigen	Non-reactive	Non-reactive
Hepatitis B Surface antibody, mIU/mL	<0.98^a^	>=12.00
Hepatitis C antibody	Non-reactive	Non-reactive
Urine toxicology		
Amphetamine, qualitative	Positive	Not Detected
Barbiturate, qualitative	Not Detected	Not Detected
Benzodiazepine, qualitative	Not Detected	Not Detected
Cannabis, qualitative	Not Detected	Not Detected
Cocaine, qualitative	Not Detected	Not Detected
Opiate, qualitative	Positive	Not Detected
Methadone, qualitative	Not Detected	Not Detected
Oxycodone, qualitative	Not Detected	Not Detected
Fentanyl, qualitative	Not Detected	Not Detected
Urinalysis (UA)		
Color	Colorless	Light yellow
Clarity	Clear	Clear
pH	7	5.0-8.0
Specific gravity	1.009	1.005-1.030
Protein	Negative	Negative
Glucose	Negative	Negative
Ketones	Negative	Negative
Bilirubin	Negative	Negative
Blood	Negative	Negative
Urobilinogen, mg/dL	<2.0	<2.0
Leukocytes	Negative	Negative
Nitrite	Negative	Negative

Approximately three months after this hospitalization, the patient was re-admitted to an outside hospital for computer cleaner inhalant intoxication. On discharge, he was again educated on stopping inhalant use and its effects and was discharged to a rehabilitation facility. He has not yet followed up with an endocrinologist for continued care.

## Discussion

Skeletal fluorosis is a metabolic bone disease endemic in developing countries due to the consumption of fluoride-contaminated water and foods [1-2,7-8]. According to the WHO, water sources with fluoride levels greater than 1.5 mg/L are unsafe [9]. Over-ingestion of fluoride may alter bone development, remodeling, and metabolism by altering osteoblast and osteoclast activity [1,10]. While the disease has been studied as a global health issue, there are a few case reports of skeletal fluorosis in industrialized countries [5,11]. This case report features the diagnostic work-up and treatment of a young male patient who presented with diffuse bone pain and was subsequently found to have skeletal fluorosis secondary to inhalant abuse from a fluoride-based computer cleaner.

Differential diagnosis of bone pain with diffusely increased sclerosis on imaging may include skeletal fluorosis, sclerosing bone dysplasia such as osteopetrosis, renal osteodystrophy, Paget disease of bone, hyperthyroidism, myeloproliferative diseases such as myelofibrosis, or marrow-infiltrating neoplasms such as lymphoma, leukemia, or osteoblastic metastasis [12]. Sclerosing bone dysplasias such as osteopetrosis, are rare genetic disorders of bone development that result in excessive bone formation. Though the diagnosis of hereditary sclerosing bone dysplasia was considered given the young age of onset of symptoms in this patient, it was considered less likely due to his lack of personal or family history of childhood fractures and lack of osseous changes in prior imaging studies. In this case, the diagnosis of skeletal fluorosis was most likely due to the patient’s history of chronic inhalant use, imaging findings, elevated serum, and urine fluoride levels, as well as the low likelihood of other conditions based on history and objective findings. It is thus crucial for clinicians to obtain a detailed medical and social history in patients presenting with diffuse bony sclerosis on diagnostic exams.

Laboratory findings of fluorosis can reveal secondary hyperparathyroidism, hypovitaminosis D, elevated alkaline phosphatase, and anemia. Secondary hyperparathyroidism may be observed due to the interference of calcium homeostasis by fluoride [13]. Replacement of vitamin D is crucial to prevent the worsening of secondary hyperparathyroidism and hypocalcemia.

Imaging findings of fluorosis can reveal osteopenia, ossification of tendons or ligaments, and diffuse sclerosis of bones, especially in the axial skeleton [4,5]. Bone and joint pain and findings of diffuse sclerosis on imaging should prompt clinicians to include skeletal fluorosis in the differential diagnosis. Elevated urine and serum fluoride as well as elevated bone fluoride content support the diagnosis of fluoride toxicity [1,2]. A dual-energy X-ray absorptiometry (DEXA) scan may show elevated Z-scores and bone mineral density [3,14]. Osteosclerosis on bone biopsy also supports the diagnosis [2]. Definitive diagnosis can be done through a quantitative bone ash fluoride analysis from bone biopsy, which is helpful in cases where laboratory and imaging studies do not clearly support the diagnosis [2]. However, as in our patient, the judicious selection of tests may reduce overall costs and provide high-value care. Due to the high diagnostic probability of skeletal fluorosis based on exposure to fluoride inhalants, imaging findings of diffuse sclerosis, and elevated serum and urine fluoride, it was determined that a bone biopsy would not provide additional diagnostic information.

The primary treatment of skeletal fluorosis is the removal of the causative agent. Cessation of fluoride exposure or consumption may reverse fluoride toxicity, improve clinical symptoms, and decrease sclerosis observed on imaging. However, laboratory and imaging abnormalities may take several years or decades to normalize due to the seven-year half-life of fluoride deposited in bone [5,15]. Symptomatic management is recommended for musculoskeletal pain and during the withdrawal phase, which may last for many weeks. Proper hydration is crucial as nephrolithiasis and hypercalciuria have been described during the skeletal unloading of fluoride [15].

A multi-disciplinary approach is recommended for the management of patients with skeletal fluorosis. This includes regularly scheduled follow-up appointments with endocrinology, primary care physician, and addiction medicine.

## Conclusions

Skeletal fluorosis may arise due to chronic recreational substance use such as computer cleaner inhalants. A thorough history and diagnostic studies must be conducted for the proper establishment of diagnosis. The involvement of multidisciplinary health professionals is crucial in the management of fluorosis, as cessation of substance use remains the sole treatment of the condition.
